# Reduction of Hydrogen Peroxide by Human Mitochondrial Amidoxime Reducing Component Enzymes

**DOI:** 10.3390/molecules28176384

**Published:** 2023-08-31

**Authors:** Sophia Rixen, Patrick M. Indorf, Christian Kubitza, Michel A. Struwe, Cathrin Klopp, Axel J. Scheidig, Thomas Kunze, Bernd Clement

**Affiliations:** 1 Department of Pharmaceutical and Medicinal Chemistry, Pharmaceutical Institute, Kiel University, 24118 Kiel, Germany; srixen@pharmazie.uni-kiel.de (S.R.); pindorf@pharmazie.uni-kiel.de (P.M.I.); mstruwe@strubio.uni-kiel.de (M.A.S.); cklopp@strubio.uni-kiel.de (C.K.); tkunze@pharmazie.uni-kiel.de (T.K.); 2 Department of Structural Biology, Zoological Institute, Kiel University, 24118 Kiel, Germany; ckubitza@strubio.uni-kiel.de (C.K.); axel.scheidig@strubio.uni-kiel.de (A.J.S.)

**Keywords:** reactive oxygen species, hydrogen peroxide, molybdenum enzyme

## Abstract

The mitochondrial amidoxime reducing component (mARC) is a human molybdoenzyme known to catalyze the reduction of various *N*-oxygenated substrates. The physiological function of mARC enzymes, however, remains unknown. In this study, we examine the reduction of hydrogen peroxide (H_2_O_2_) by the human mARC1 and mARC2 enzymes. Furthermore, we demonstrate an increased sensitivity toward H_2_O_2_ for HEK-293T cells with an *MTARC1* knockout, which implies a role of mARC enzymes in the cellular response to oxidative stress. H_2_O_2_ is a reactive oxygen species (ROS) formed in all living cells involved in many physiological processes. Furthermore, H_2_O_2_ constitutes the first mARC substrate without a nitrogen–oxygen bond, implying that mARC enzymes may have a substrate spectrum going beyond the previously examined *N*-oxygenated compounds.

## 1. Introduction

The human mARC enzyme was first described in 2006 as the third component of the *N*-reducing complex together with hemoprotein cytochrome b5 (Cyb5B) and flavoprotein cytochrome b5 reductase (Cyb5R3) [1]. Together with these two electron carrier proteins, mARC enzymes reduce various *N*-hydroxylated substrates like amidoximes, *N-*hydroxy-guanidines, hydroxylamines, *N*-oxides or hydroxamic acids [2]. mARC utilizes a Mo-molybdopterin cofactor (Moco), the coordination of the catalytic molybdenum ion being very similar to that observed in sulfite oxidase (SO) [3,4] despite not sharing many other characteristics with SO. Thus, mARC enzymes are classified as part of a separate, new family of molybdenum enzymes, the MOSC domain family [5,6]. All mammalian genomes encode two paralogues of mARC: mARC1 and mARC2 (gene names *MTARC1*, *MTARC2*) [4].

Hydrogen peroxide (H_2_O_2_) is the major reactive oxygen species (ROS) in eukaryotic cells and some 37 human enzymes are known to generate H_2_O_2_ by the two-electron reduction of dioxygen [7]. Interestingly, among these H_2_O_2_-producing enzymes are the eukaryotic molybdenum enzymes xanthine oxidase (XO) [8], aldehyde oxidase (AO) [9] and sulfite oxidase (SO) [10].

While high concentrations of H_2_O_2_ cause oxidative damage to cells, it is considered to be a physiologically relevant signaling molecule at lower concentrations. These different effects of H_2_O_2_ have been reviewed in detail elsewhere [7].

Various cell compartments contain H_2_O_2_-degrading enzymes, ensuring tight regulation of H_2_O_2_ concentrations. Examples are catalase or myeloperoxidase, which, due to their high K_M_ values, are suitable for degrading high H_2_O_2_ concentrations, e.g., in peroxisomes [11,12]. Other enzymes like GPx1 or peroxiredoxins act at much lower H_2_O_2_ concentrations characteristic for their respective cell compartments [13,14].

Until now, no H_2_O_2_-degrading enzyme has been identified in the outer mitochondrial membrane (OMM), where mARC enzymes are localized [15].

The mARC enzyme system is known best for its reductive activity toward *N*-oxygenated compounds. However, some studies have shown links between mARC and ROS. For example, the common mARC1 p.A165T variant is associated with higher levels of lipid peroxidation, while the total antioxidant activity (TAA) in serum and expression of catalase are increased [16].

In this work, we present the NADH-dependent degradation of H_2_O_2_ by the human mARC1 and mARC2 enzymes in concert with their electron carriers Cyb5B and CYB5R3 using recombinant proteins. We go on to show the effect of an *MTARC1* knockout on the viability of HEK-293T cells in the presence of high external H_2_O_2_ concentrations. 

## 2. Results

### 2.1. Molybdenum-Containing mARC1 and mARC2 Both Reduce H_2_O_2_

**Figure 1:**
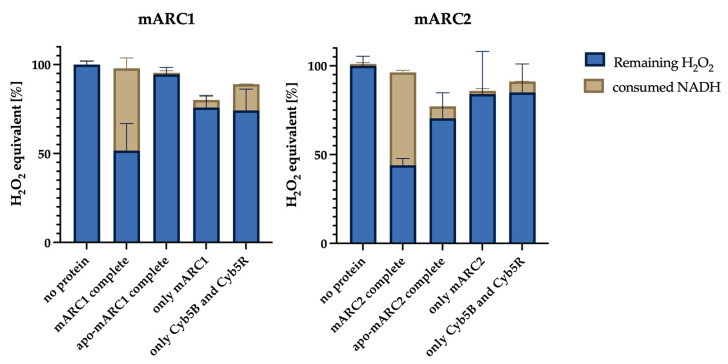
Stacked bar-chart representing the consumption of NADH (brown columns) on top of the residual amount of H_2_O_2_ (blue columns) for mARC1 (panel A) and mARC2 (panel B). The individual setups compared to each other are (from left to right): no protein—assay containing only NADH and H_2_O_2_ but no enzymes; mARC1/2 complete—contains mARC1 or mARC2 and both electron carrier proteins; apo-mARC1 complete—same as before but with molybdenum-free apo-mARC; only mARC1/2—just mARC1/2, but no electron carriers; only Cyb5B and Cyb5R—only electron carriers but no mARC enzymes.

To assess the reduction of H_2_O_2_ by mARC enzymes, we compared the NADH consumption, measured in the NADH assay, and the amount of residual H_2_O_2_, quantified by the fluorometric assay, for several different setups. Importantly, extensive control reactions were examined to unambiguously identify the effect of molybdenum-containing mARC enzymes. The results from these assays are visualized in Figure 1. Note that different H_2_O_2_ concentrations (50 µM for mARC1 and 80 µM for mARC2) were used due to different stabilities of the enzymes toward high H_2_O_2_ concentrations. 

It is clearly observed that, for both mARC1 and mARC2, the by far greatest depletion of NADH is observed when the complete, reconstituted mARC1/2 enzyme systems are used. Correspondingly, in these reactions, the lowest concentrations of residual H_2_O_2_ were found, which confirms that NADH consumed by the mARC enzyme system does in fact reduce H_2_O_2_. The control reactions indicate that only holo-mARC enzymes with a molybdopterin prosthetic group can reduce H_2_O_2_ in concert with Cyb5B and Cyb5R3.

### 2.2. Kinetics of mARC-Dependent H_2_O_2_ Reduction

**Figure 2:**
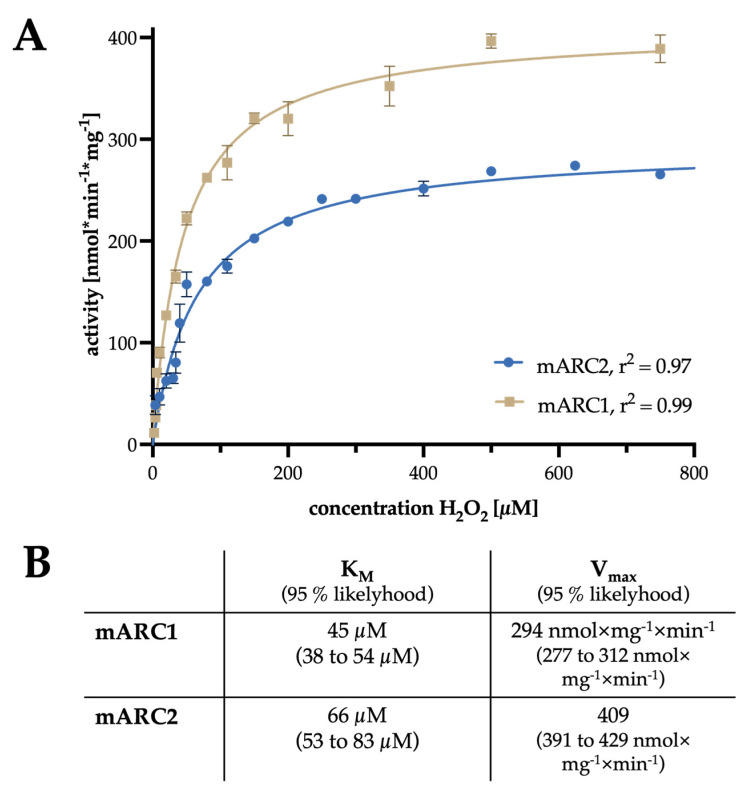
(**A**) Kinetic profile of mARC1- and mARC2-catalyzed H_2_O_2_ reduction. (**B**) Kinetic parameters obtained from fitting to the Michaelis–Menten equation. Values in parentheses indicate the 95% likelihood interval for K_M_ and V_max_.

Both mARC1 and mARC2 display Michaelis–Menten kinetics for H_2_O_2_ reduction, as is shown in Figure 2. The turnover rates and K_M_ values for H_2_O_2_ reduction by mARC1 and mARC2 are comparable, with mARC1 showing a slightly lower K_M_ but higher turnover rates.

Using our recently established fluorescence-based high-throughput assay [17], we were able to measure very similar conversion rates for the mARC-catalyzed reduction of hydrogen peroxide.

### 2.3. MTARC1 Knockout Decreases Cell Viability in Presence of H_2_O_2_

**Figure 3:**
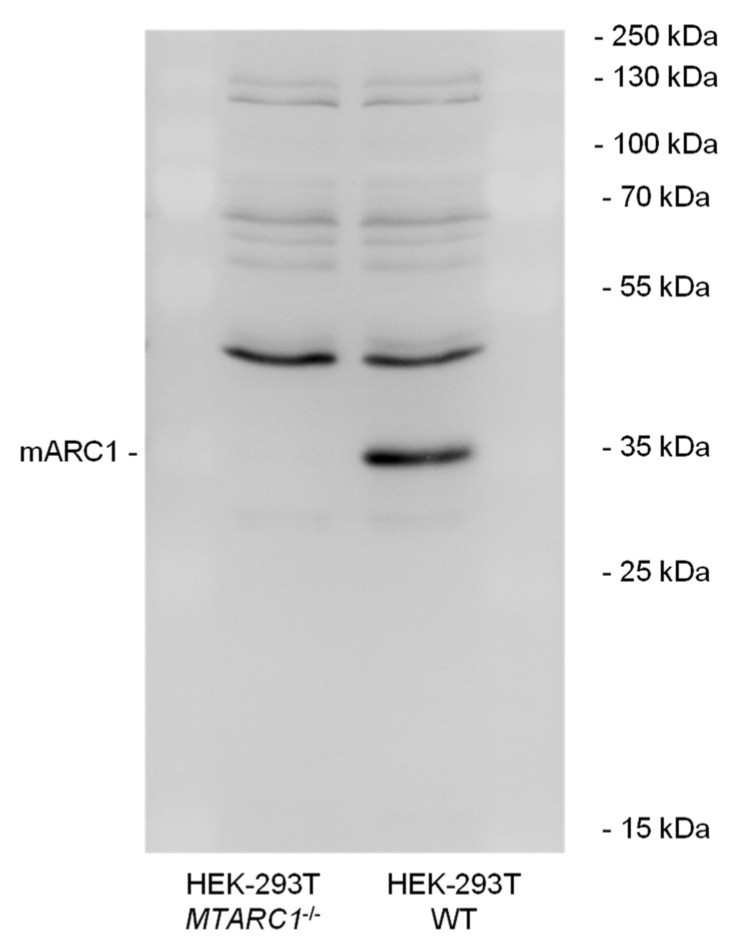
Protein production to verify *MTARC1* KO. *MTARC1*^−/−^ and WT cells were lysed, 36 µg of protein was applied per lane and Western blot analyses were performed using an anti-mARC1 antibody.

To determine whether or not the reduction of H_2_O_2_ by recombinant mARC enzymes is relevant in cellulo, we examined the impact of different H_2_O_2_ concentrations on cell viability using an HEK-293T-based knockout model. Since HEK-293T cells express only very low levels of mARC2, the *MTARC1* knockout results in cells practically devoid of mARC activity (mARC2 expression levels do not increase to compensate the *MTARC1* knockout. The knockout was shown to be effective on the protein level by Western blot analysis (Figure 3). Before incubation with H_2_O_2_, some cells were treated with buthionine sulfoximine (BSO), an inhibitor of glutathione synthesis.

**Figure 4:**
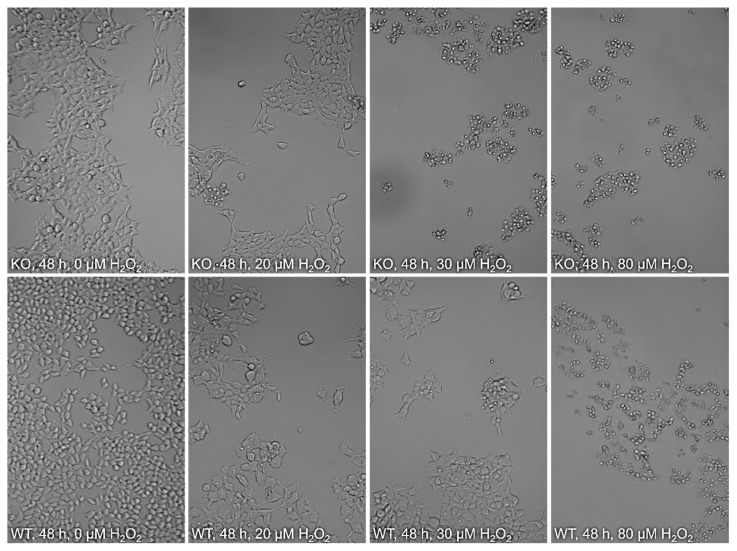
Cytotoxicity of H_2_O_2_ to HEK-293T *MTARC1*-KO and WT cells. Cells were seeded onto 96-well plates and incubated with medium containing 0.3 mM BSO for 16 h, followed by incubation with medium containing 20–80 µM H_2_O_2._ After 48 h of incubation, cell morphology was examined microscopically. At 0–20 µM H_2_O_2_, both WT and KO cells have very similar morphologies. However, when the H_2_O_2_ concentration is increased to 30 µM, morphology of KO cells changes drastically, whereas the WT cells look largely unaffected. At 80 µM, both cell lines display strong morphological differences, resembling the changes already seen at 30 µM for KO cells.

**Figure 5:**
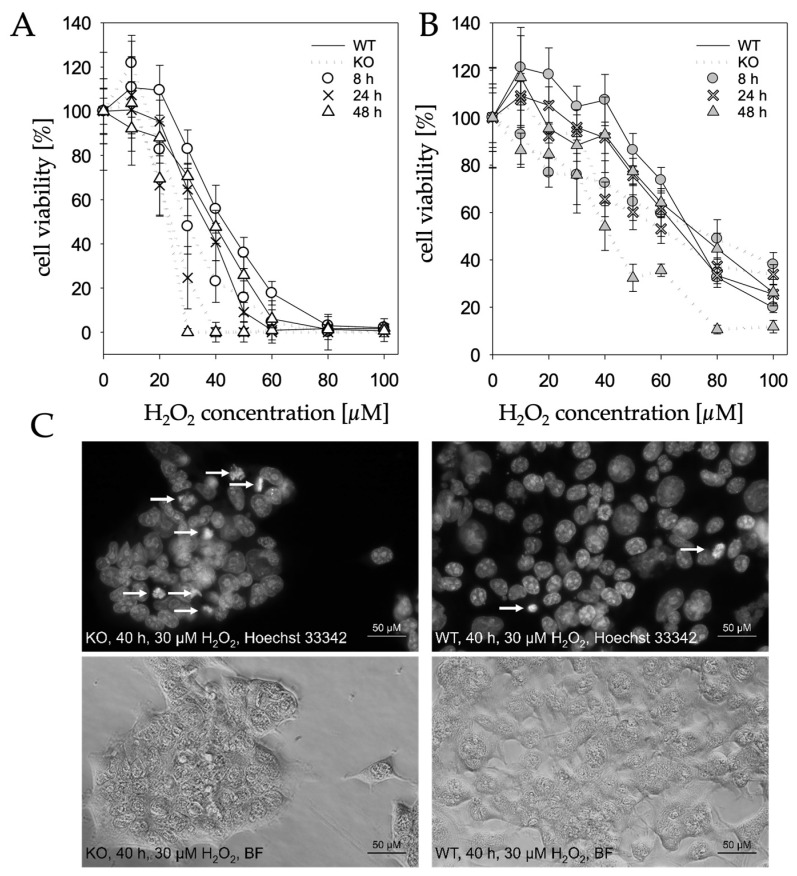
Cell viability and Hoechst 33342 staining. Cells were treated with different concentrations of H_2_O_2_ and examined after 8 h, 24 h, 48 h and 72 h by resazurin assay. Cells treated with medium without supplemented H_2_O_2_ were defined as 100% viability. (**A**) Cell viability after prior treatment with 0.3 mM BSO; (**B**) cell viability without prior treatment with BSO; (**C**) fluorescence and bright film microscopy of HEK-293T *MTARC1* KO and WT cells in a 40× objective magnification. Cells were treated for 48 h with 30 µM H_2_O_2_. The white arrows mark cell nuclei with clear chromatin condensation.

Differences seen between wildtype and knockout cells are already reflected by cell morphology. A changed cell morphology induced by H_2_O_2_, which can be observed in KO cells at 20 µM, only occurs in WT cells at 30 µM, while the KO cells at 30 µM can hardly be considered morphologically alive (Figure 4). Furthermore, Hoechst staining revealed that knockout cells treated with 30 µM H_2_O_2_ had higher nuclear condensation and thereby an increased apoptosis rate compared to wildtype cells (Figure 5C).

This observation was confirmed in a resazurin-based cell viability assay. While low concentrations of H_2_O_2_ do not appear to have a negative influence on cell viability in either WT or KO cells, when increasing H_2_O_2_ concentrations above 10 µM, WT and KO cells clearly show divergence, with the viability of WT cells being significantly higher (Figure 5A,B). A decreased viability of knockout cells can already be observed after 8 h and only becomes even more pronounced after longer incubation periods. After 48 h, KO cells are not viable at 30 µM H_2_O_2_, whereas the same is observed with WT cells at a concentration of 80 µM H_2_O_2_. At a concentration of 30 µM, the viability of the WT cells is still approx. 70%.

These findings show that H_2_O_2_ degradation by the mARC1 enzyme does occur in cell culture, and it has a measurable effect on cell physiology at high extracellular H_2_O_2_ concentrations.

**Figure 6:**
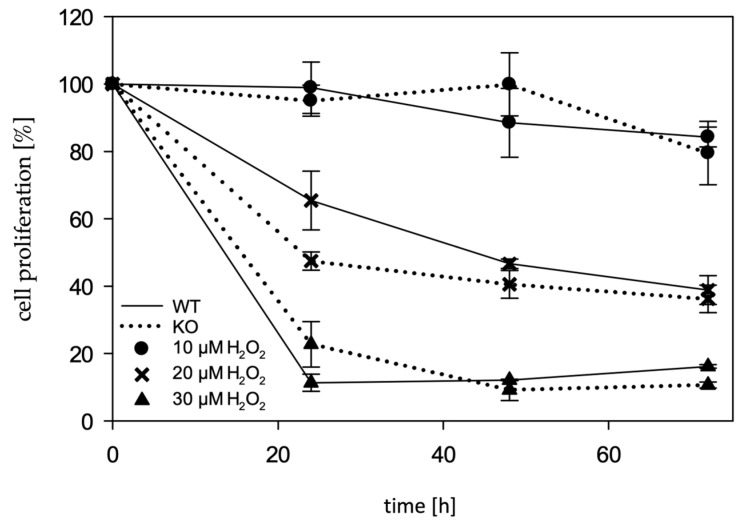
Cell proliferation. Cells were treated with different concentrations (10 µM, 20 µM, 30 µM) of H_2_O_2_. After 24 h, 48 h and 72 h, the cell number was determined on the ImageXpress^®^ (λ_ex_: 358, λ_em_: 461) (Molecular Devices, LLC., San Jose, CA, USA).

Further, an influence on cell proliferation could be observed. Extracellular concentrations of 10 µM H_2_O_2_ showed no impairment on cell proliferation. An extracellular H_2_O_2_ concentration of 20 µM did lead to impaired cell proliferation: after 24 h, the proliferation of both WT and KO cells was decreased compared to control cells without H_2_O_2_ treatment. This impairment on cell proliferation was more pronounced in mARC1-deficient cells, where, after 24 h, only 50% could be counted compared to cells treated with the medium only; thus, in purely arithmetical terms, no cell division of the KO cells had taken place in the last 24 h. The number of WT cells was reduced to approx. 70%. After 72 h, both WT and KO cells were reduced to approx. 35%. At a H_2_O_2_ concentration of 30 µM, no measurable cell division occurred in either the KO or WT cells (Figure 6).

## 3. Discussion

The study presented here identifies H_2_O_2_ as a new substrate for the human mARC1 and mARC2 proteins. The degradation of H_2_O_2_ was demonstrated in vitro with recombinant enzymes and confirmed in a more complex environment by in cellulo knockout studies. Thus, for the first time, a reduction of *O-O* bonds by the mARC enzyme system is described. Molybdenum enzymes like mARC typically characterize two-electron transfer reactions; therefore, the product of this reaction is likely water.

While we cannot at this point conclude that H_2_O_2_ or other reactive oxygen species are the physiological substrate of eukaryotic mARC enzymes, this finding is important nonetheless. Compounds with *O-O* bonds are a completely novel group of potential mARC substrates that have not previously been studied. So far, all mARC-catalyzed reactions described in the literature are *N*-reductions cleaving *N-O* bonds [18]. 

The turnover rates of the mARC-catalyzed H_2_O_2_ reduction are relatively low compared to other H_2_O_2_-degrading enzymes. It should be noted, however, that turnover rates determined with the soluble recombinant proteins without their OMM-anchoring sequences are typically much lower for human mARC enzymes compared with proteins isolated from organ homogenates [19]. Thus, in vivo H_2_O_2_-reducing activities of human mARC enzymes can be expected to be significantly higher than the values reported here. On another note, the K_M_ values for H_2_O_2_ reduction by recombinant human mARC proteins (approx. 50 µM) are very low compared to those for the well-studied *N*-hydroxylated compounds. 

Hydrogen peroxide has fundamentally important functions in humans. Depending on the intracellular concentration, it initiates, inter alia, cell proliferation, cell shaping, migration and angiogenesis [20,21,22]. On the other hand, the accumulation of higher concentrations of hydrogen peroxide and other ROS leads to oxidative stress, a condition of imbalance between pro-oxidants and antioxidants. ROS pass through cell membranes and cause oxidative damage to lipids, proteins and DNA, as well as mitochondrial dysfunction, all of which can lead to the loss of essential cell functions and initiate the caspase-mediated apoptosis pathway [23,24,25].

In this study, a coupled enzyme assay was established. Two parameters were measured: the amount of NADH oxidized by the mARC-mediated reduction and, to confirm the results, the remaining concentration of hydrogen peroxide. Thus, it was shown that both mARC proteins can reduce H_2_O_2_. 

Considering the enzyme kinetics of this reaction, it is striking that the K_M_ values of both mARC proteins are remarkably lower when compared to well-known H_2_O_2_-depleting enzymes such as catalase or peroxiredoxin [26,27]. 

An in cellulo *MTARC1* knockout model was generated and established to verify whether the absence of mARC1 leads to cellular impairment upon exposure to H_2_O_2_. A significantly reduced cell physiology of mARC1-deficient cells and thus a higher sensitivity toward H_2_O_2_ compared to corresponding WT cells could be observed. Also, higher apoptosis levels and lower cell viability levels were seen. These findings could be confirmed by light and fluorescence microscopy showing altered cell morphology and declined nuclear condensation. While the H_2_O_2_ concentrations used in our cell culture experiments certainly exceed those expected in vivo, it is still possible that the regulation of ROS is a physiological function of mARC enzymes. 

The complex mechanisms of hydrogen peroxide regulation with a large number of enzymes in various cell organelles demonstrate the need for different approaches to control the intracellular concentration. While major hydrogen peroxide transforming enzymes like catalase, GPx or peroxiredoxins are present in the endoplasmic reticulum, cytosol, nucleus, peroxisomes, the intermembrane space (IMS), inner mitochondrial membrane (IMM) and mitochondrial matrix, mARC stands out through its localization at the OMM [13,15,28,29,30], although there are some reports about GPx also being localized at the OMM [31]. 

mARC might thus be involved in protecting the OMM from ROS. Since ROS are formed in high concentrations in the IMS and the cytosol by different enzymes, a protective mechanism for the undesired oxygenation of the OMM—for example, against lipid peroxidation to prevent oxidative stress and mitochondrial dysfunction—is conceivable. This was also described earlier for GPx-4 at the IMM [28]. There are also some known enzymes at the OMM, such as the monoamine oxidase MAO, that form hydrogen peroxide as a secondary product [28]. It is thus also possible that mARC influences the free diffusion between IMS and cytosol diffusion and the transport of H_2_O_2_ through voltage-dependent anion channels (VDACs) and peroxiporins due to its high affinity to H_2_O_2_.

Kagan and colleagues highlighted the significance of ROS for apoptosis by identifying the releasing pathway of proapoptotic factors from the OMM. A H_2_O_2-_dependent cardiolipin-specific peroxidase activity of cytochrome c is required for the permeabilization of the OMM, demonstrating again the significance of hydrogen peroxide regulation in the OMM for critical cell processes [32]. Also, H_2_O_2_ is formed in the peroxisomal β-oxidation of fatty acids [33]. Various studies in mice and rats also suggest the possibility of a dual localization of mARC in mitochondria and peroxisomes [34,35]; thus, this colocalization could suggest that mARC has a regulatory function in hydrogen peroxide and antioxidant metabolism in peroxisomes as well. 

In conclusion, the reduction of H_2_O_2_ by mARC is certainly very interesting, as it indicates that the spectrum of substrates that these enzymes can reduce could go far beyond the previously studied *N*-oxygenated substrates. The in cellulo studies confirm that H_2_O_2_ is also reduced by the native mARC enzyme in its cellular context. We do not claim that H_2_O_2_ or other reactive oxygen species are the physiological substrates of mARC. An involvement in the cellular regulation of H_2_O_2_ is conceivable, but data available on this point are not sufficient to claim this to be the enzymes’ function. Due to their involvement in liver disease, mARC enzymes have recently gained much attention. However, it remains unknown what the physiological function of mARC actually is and how exactly it exerts its influence on lipid metabolism and liver disease. In the future, a search for mARC’s physiological substrate that might not have previously been associated with mARC should be considered. 

## 4. Materials and Methods

### 4.1. Protein Sources

Recombinant human mARC1, mARC2, Cyb5B and Cyb5R3 were expressed in *Escherichia coli* (*E. coli*) and purified by column chromatography, essentially as described previously [36]. For mARC1 and mARC2 with bound molybdopterin cofactor (*holo*-mARC1/2), the *E. coli* TP1000 strain [37] was used. Proteins without molybdopterin were expressed in RK5202 [38]. Protein concentrations were determined using the Pierce BCA Protein Assay Kit (Thermo Fisher Scientific, Waltham, MA, USA) with bovine serum albumin for calibration. Loading of Cyb5B with heme and Cyb5R3 with flavin adenine dinucleotide (FAD) was quantified as published [36]. 

### 4.2. Photometric Assay

Reduction of H_2_O_2_ by the reconstituted mARC enzyme system was assayed using the previously published protocol [2]. Reactions contained 7.5 µg (224 pmol) of either mARC1 or mARC2, 3.5 µg (210 pmol) Cyb5B and 0.08 µg (2.4 pmol) Cyb5R3 and 200 µM NADH in 20 mM Na-MES buffer, pH 6.0. The total reaction volume was 300 µL. Consumption of NADH at 37 °C was monitored by recording the absorption spectrum from 300 to 400 nm in 15 s intervals. The reaction was stopped by heating 200 µL of the incubation mix to 95 °C for 5 min in a water bath. Turnover rates were calculated through the change in absorption at 340 nm over a timespan of 2 min. Kinetic parameters were determined by fitting the Michaelis–Menten equation to the turnover rates at different H_2_O_2_ concentrations in GraphPad Prism 9.5.1. All measurements were performed in triplicate.

### 4.3. Fluorometric Activity Assay

Alternatively, the enzyme activity was assayed by monitoring NADH consumption through a recently established fluorometric protocol [17]. Briefly, time-dependent change in NADH fluorescence (λ_ex_ = 340 nm; λ_em_ = 365 nm) was monitored with a TECAN Infinite 200 M Pro plate reader. The reaction volume was 50 µL. Assays contained 193 pmol (=6.5 µg) *h*mARC-1, 65 pmol *h*Cyb5B (heme), 6.5 pmol *h*Cyb5R3 (FAD), 0.2 mM of NADH and the substrate to be tested in 20 mM Na-MES buffer, pH 6.0. The reaction mixtures containing all components except Cyb5R3 were pre-incubated at 37 °C for 3 min. The reactions were started by adding Cyb5R3, and NADH fluorescence was recorded for 15 min at 37 °C. BAO was always used in parallel as a reference substrate.

### 4.4. Peroxide Assay

To confirm degradation of H_2_O_2_ by the mARC enzyme system, residual H_2_O_2_ concentrations were quantified using a fluorometric peroxidase assay [39]. Samples from the photometric activity assays were cooled on ice for 1 min and then pre-incubated at 37 °C for 2 min. Then, 10 µL of 20 mM Na-MES buffer, pH 6.0, 30 µL of 20 mM 4-hydroxyphenylacetic acid and 30 µL of a 30 µg/mL horseradish peroxidase solution were added and incubation at 37 °C was continued for 10 min. Afterwards, 10 µL of 10 M NaOH and 850 µL distilled water were added. A total of 150 µL was transferred to Perkin Elmer quartz SUPRASIL cuvettes. Fluorescence spectra from 340 to 450 nm were measured in a Perkin Elmer LS 55 Fluorescence Spectrometer using an excitation wavelength of 320 nm. The peak at 408 nm was used for evaluation. Correlation between the intensity of this peak and the H_2_O_2_ concentration was proven using a calibration curve. 

### 4.5. Molecular Biology

Knockout of the *MTARC1* gene in HEK-293T cells was achieved by the CRISPR-Cas9 method [40]. A sequence encoding sgRNA for sgRNA addressing exon 2 of the *MTARC1* gene (5′-GTGGCCAAAACCGAACACTAGT-TGG-3′, PAM sequence underlined) was cloned into the Esp3I site of the plentiCRISPRv2 plasmid (Addgene #49535) using standard cloning methods [41]. Correct insertion of the sgRNA-encoding sequence was confirmed by Sanger sequencing using the primer 5′-GAGGGCCTA-TTTCCCATGATTCC-3′.

### 4.6. Mammalian Cell Culture

Human embryonic kidney cells (HEK-293T) were grown in Dulbecco’s Modified Eagle Medium (DMEM) supplemented with 10% fetal calf serum (FCS) in a humidified incubator at 37 °C in presence of 5% CO_2_. HEK-293T cells were verified by SNP analysis and confirmed to be mycoplasma-free. 

For transfection, target cells were seeded at 2 × 10^5^ cells/well in a 6-well plate. Twenty-four hours after seeding, the cultivation medium was replaced with DMEM containing 2% FCS. The transfection mix consisted of 100 µL Opti-MEM, 1 µg DNA (plentiCRISPRv2 containing the sgRNA sequence) and 3 µL Lipofectamine 2000^®^ (Thermo Fisher Scientific, Waltham, MA, USA). Medium was exchanged for DMEM incl. 10% FCS after 6 h. After further 18 h, medium was replaced again by DMEM incl. 10% FCS, supplemented with 2.5 µg/mL puromycin. Cells were cultivated and selected in puromycin-containing medium for 6 days.

HEK-293T KO lines were isolated by serial dilution in 96-well plates (0.5 cells/well). After three weeks of expansion, DNA was isolated with the peqGOLD microspin tissue DNA kit (VWR, Darmstadt, Germany), the region of interest was amplified by PCR and the knockout was validated by Sanger sequencing. Primers for both amplification of the gene region of interest as well as sequencing were 5′-AAGCTCCTCCAGGGTCTGGCTTC-3′ and reverse 5′-CGACCTGCCCTTTCCTTACCTGC-3′. 

For immunoblot analysis, cells were detached using ice-cold Dulbecco’s PBS (DPBS), centrifuged, and resuspended in NP-40 lysis buffer (containing 150 mM NaCl, 1% (*v*/*v*) Nonidet P-40, 50 mM Tris). After 30 min shaking at 4 °C, the lysate was centrifuged again and the protein concentration in the supernatant was quantified using the Pierce BCA Protein Assay Kit (Thermo Fisher Scientific). 

### 4.7. SDS-PAGE and Immunoblotting

Samples containing 36 µg of total protein were separated by SDS-PAGE on hand-cast MiniProtean gels supplemented with 0.5% trichloroethanol (TCE) (*v*/*v*) (Bio-Rad, Hercules, CA, USA) according to standard protocols. TCE was used as an unspecific protein staining. It reacts with tryptophan residues of the proteins under UV radiation for 5 min to a fluorescent product [42]. After electrophoresis, proteins were transferred onto Hybond-P polyvinylidene fluoride membranes (GE Healthcare, Chicago, IL, USA). The membranes were blocked in TRIS-buffered saline containing 0.1% Tween 20 (TBST) and 5% milk powder, incubated with primary antibodies and washed with TBST. Antibodies used were anti-mARC1 (Abgent, San Diego, CA, USA; AP9754c, 1:1000 dilution) and a horseradish peroxidase-conjugated goat anti-rabbit antibody (Jackson ImmunoResearch Laboratories, Ely, UK). Fluorescence and chemiluminescence were detected on a ChemoStar Touch ECL and Fluorescence Imager (Intas Science Imaging, Göttingen, Germany).

### 4.8. Viability Assay

Both wildtype and *MTARC1* knockout HEK-293T cells were seeded at 3000 cells per well into 96-well plates containing 80 µL DMEM (with 10% FCS). Twenty-four hours after seeding, adherent cells were incubated with medium containing 0.3 mM BSO for 16 h, followed by incubation with medium containing 0, 10, 20, 30, 40, 50, 60, 80, 100 µM H_2_O_2_. Cell viability was assayed using a water-soluble resazurin assay (Sigma Aldrich) after 8, 24 and 48 h. A total of 11 µL of a 0.01% resazurin solution in PBS was added directly to the culture medium (10% of the culture medium volume, 0.001% resazurin). After 180 min incubation at 37 °C, the amount of converted resazurin was measured fluorometrically (λ_ex_ = 530 nm, λ_em_ = 590 nm) in a spark^®^ multimode microplate reader (Tecan Trading AG, Männedorf, Switzerland). The fluorescence measured for cells treated with 0 µM H_2_O_2_ were defined as 100% viability. mARC1 itself does not significantly contribute to resazurin reduction. 

### 4.9. Proliferation Assay

To determine the influence of H_2_O_2_ on cell proliferation, the same number of cells were seeded in black 96-well microtiter plates with transparent bottom. Cells were fixed and stained with Hoechst 33342 after further 24 h, 48 h and 72 h of incubation with 10 µM, 20 µM and 30 µM H_2_O_2_. For this purpose, 50 µL of 100 µL culture medium was removed and replaced by 50 µL of an 8% PFA, 0.002% Hoechst 33342 solution in DPBS. After 10 min of incubation at RT, the supernatant was completely removed, and each well was washed twice with DPBS. Cells were overcoated with DPBS and counted on the ImageXpress^®^ (λ_ex_: 358, λ_em_: 461) (Molecular Devices, LLC., San Jose, CA, USA).

### 4.10. Microscopy

After 40 h of incubation at 37 °C, cells were imaged live at 20× objective magnification on an Olympus CK2 microscope. For Hoechst 33342 staining, 1500 cells were seeded onto 96-well half-area black microplates and incubated with H_2_O_2_ as described before. Cells were fixed with 8% formaline in PBS for 10 min at room temperature and simultaneously stained with Hoechst 33342. After staining, wells were rinsed twice with PBS to remove any remaining dye. Apoptotic cells were observed under a fluorescence microscope at 40× objective magnification (Olympus, Tokyo, Japan).

## Data Availability

Not applicable.

## References

[1] Havemeyer A., Bittner F., Wollers S., Mendel R., Kunze T., Clement B. (2006). Identification of the missing component in the mitochondrial benzamidoxime prodrug-converting system as a novel molybdenum enzyme. J. Biol. Chem..

[2] Indorf P., Kubitza C., Scheidig A., Kunze T., Clement B. (2019). Drug metabolism by the Mitochondrial Amidoxime Reducing Component (mARC): Rapid assay and identification of new substrates. J. Med. Chem..

[3] Rajapakshe A., Astashkin A.V., Klein E.L., Reichmann D., Mendel R.R., Bittner F., Enemark J.H. (2011). Structural studies of the molybdenum center of mitochondrial amidoxime reducing component (mARC) by pulsed EPR spectroscopy and ^17^O-labeling. Biochemistry.

[4] Ott G., Havemeyer A., Clement B. (2015). The mammalian molybdenum enzymes of mARC. J. Biol. Inorg. Chem..

[5] Kubitza C., Bittner F., Ginsel C., Havemeyer A., Clement B., Scheidig A.J. (2018). Crystal structure of human mARC1 reveals its exceptional position among eukaryotic molybdenum enzymes. Proc. Natl. Acad. Sci. USA.

[6] Gladyshev V.N., Zhang Y., Hille R., Schulzke C., Kirk M.L. (2016). Abundance, Ubiquity and Evolution of Molybdoenzymes. Molybdenum and Tungsten Enzymes: Biochemistry.

[7] Sies H. (2017). Hydrogen peroxide as a central redox signaling molecule in physiological oxidative stress: Oxidative eustress. Redox Biol..

[8] Kelley E.E., Khoo N.K., Hundley N.J., Malik U.Z., Freeman B.A., Tarpey M.M. (2010). Hydrogen peroxide is the major oxidant product of xanthine oxidase. Free Radic. Biol. Med..

[9] Garrido C., Leimkühler S. (2021). The Inactivation of Human Aldehyde Oxidase 1 by Hydrogen Peroxide and Superoxide. Drug Metab. Dispos..

[10] Hänsch R., Lang C., Riebeseel E., Lindigkeit R., Gessler A., Rennenberg H., Mendel R.R. (2006). Plant sulfite oxidase as novel producer of H_2_O_2_: Combination of enzyme catalysis with a subsequent non-enzymatic reaction step. J. Biol. Chem..

[11] But P.G., Fomina V.A., Murav’ev R.A., Rogovin V.V. (2003). Myeloperoxidase from Neutrophil Peroxisomes. Biol. Bull. Russ. Acad. Sci..

[12] Deisseroth A., Dounce A.L. (1970). Catalase: Physical and chemical properties, mechanism of catalysis, and physiological role. Physiol. Rev..

[13] Cox A.G., Pearson A.G., Pullar J.M., Jönsson T.J., Lowther W.T., Winterbourn C.C., Hampton M.B. (2009). Mitochondrial peroxiredoxin 3 is more resilient to hyperoxidation than cytoplasmic peroxiredoxins. Biochem. J.

[14] Brigelius-Flohé R., Maiorino M. (2013). Glutathione peroxidases. Biochim. Biophys. Acta.

[15] Klein J.M., Busch J.D., Potting C., Baker M.J., Langer T., Schwarz G. (2012). The Mitochondrial Amidoxime-Reducing Component (mARC1) is a novel signal-anchored protein of the outer mitochondrial membrane. J. Biol. Chem..

[16] Janik M.K., Smyk W., Kruk B., Szczepankiewicz B., Gornicka B., Lebiedzinska-Arciszewska M., Potes Y., Simoes I.C.M., Weber S.N., Lammert F. (2021). *MARC1* p.A165T variant is associated with decreased markers of liver injury and enhanced antioxidant capacity in autoimmune hepatitis. Sci. Rep..

[17] Klopp C., Struwe M.A., Plieth C., Clement B., Scheidig A.J. (2023). New Design of an Activity Assay Suitable for High-Throughput Screening of Substrates and Inhibitors of the Mitochondrial Amidoxime Reducing Component (mARC). Anal. Chem..

[18] Clement B., Struwe M.A. (2023). The History of mARC. Molecules.

[19] Chamizo-Ampudia A., Galvan A., Fernandez E., Llamas A. (2011). The *Chlamydomonas reinhardtii* molybdenum cofactor enzyme crARC has a Zn-dependent activity and protein partners similar to those of its human homologue. Eukaryot. Cell.

[20] Cordeiro J.V., Jacinto A. (2013). The role of transcription-independent damage signals in the initiation of epithelial wound healing. Nat. Rev. Mol. Cell Biol..

[21] Burdon R.H., Rice-Evans C. (1989). Free radicals and the regulation of mammalian cell proliferation. Free Radic. Res. Commun..

[22] Sies H., Jones D.P. (2020). Reactive oxygen species (ROS) as pleiotropic physiological signalling agents. Nat. Rev. Mol. Cell Biol..

[23] Janssen-Heininger Y.M., Mossman B.T., Heintz N.H., Forman H.J., Kalyanaraman B., Finkel T., Stamler J.S., Rhee S.G., van der Vliet A. (2008). Redox-based regulation of signal transduction: Principles, pitfalls, and promises. Free Radic. Biol. Med..

[24] Chiurchiù V., Maccarrone M. (2011). Chronic inflammatory disorders and their redox control: From molecular mechanisms to therapeutic opportunities. Antioxid. Redox Signal..

[25] Huang B., Liang J.J., Zhuang X., Chen S.W., Ng T.K., Chen H. (2018). Intravitreal Injection of Hydrogen Peroxide Induces Acute Retinal Degeneration, Apoptosis, and Oxidative Stress in Mice. Oxid. Med. Cell. Longev..

[26] Kehrer J.P., Robertson J.D., Smith C.V., McQueen C.A. (2010). 1.14—Free Radicals and Reactive Oxygen Species. Comprehensive Toxicology.

[27] Chandrashekar R., Tsuji N., Morales T.H., Carmody A.B., Ozols V.O., Welton J., Tang L. (2000). Removal of hydrogen peroxide by a 1-cysteine peroxiredoxin enzyme of the filarial parasite Dirofilaria immitis. Parasitol. Res..

[28] Riemer J., Schwarzländer M., Conrad M., Herrmann J.M. (2015). Thiol switches in mitochondria: Operation and physiological relevance. Biol. Chem..

[29] Florian S., Wingler K., Schmehl K., Jacobasch G., Kreuzer O.J., Meyerhof W., Brigelius-Flohé R. (2001). Cellular and subcellular localization of gastrointestinal glutathione peroxidase in normal and malignant human intestinal tissue. Free Radic. Res..

[30] Frederick S.E., Newcomb E.H. (1969). Cytochemical localization of catalase in leaf microbodies (peroxisomes). J. Cell Biol..

[31] Chatzi A., Manganas P., Tokatlidis K. (2016). Oxidative folding in the mitochondrial intermembrane space: A regulated process important for cell physiology and disease. Biochim. Biophys. Acta.

[32] Kagan V.E., Tyurin V.A., Jiang J., Tyurina Y.Y., Ritov V.B., Amoscato A.A., Osipov A.N., Belikova N.A., Kapralov A.A., Kini V. (2005). Cytochrome c acts as a cardiolipin oxygenase required for release of proapoptotic factors. Nat. Chem. Biol..

[33] Tsukamoto H., Horne W., Kamimura S., Niemelä O., Parkkila S., Ylä-Herttuala S., Brittenham G.M. (1995). Experimental liver cirrhosis induced by alcohol and iron. J. Clin. Investig..

[34] Islinger M., Luöers G.H., Li K.W., Loos M., Voölkl A. (2007). Rat Liver Peroxisomes after Fibrate Treatment: A Survey using quantitative mass spectrometry. J. Biol. Chem..

[35] Wiese S., Gronemeyer T., Ofman R., Kunze M., Grou C.P., Almeida J.A., Eisenacher M., Stephan C., Hayen H., Schollenberger L. (2007). Proteomics Characterization of Mouse Kidney Peroxisomes by Tandem Mass Spectrometry and Protein Correlation Profiling. Mol. Cell. Proteom..

[36] Wahl B., Reichmann D., Niks D., Krompholz N., Havemeyer A., Clement B., Messerschmidt T., Rothkegel M., Biester H., Hille R. (2010). Biochemical and spectroscopic characterization of the human Mitochondrial Amidoxime Reducing Components hmARC-1 and hmARC-2 suggests the existence of a new molybdenum enzyme family in eukaryotes. J. Biol. Chem..

[37] Palmer T., Santini C.-L., lobbi-Nivol C., Eave D.J., Boxer D.H., Giordano G. (1996). Involvement of the narJ and mob gene products in distinct steps in the biosynthesis of the molybdoenzyme nitrate reductase in *Escherichia coli*. Mol. Biol..

[38] Stewart V., MacGregor C.H. (1982). Nitrate Reductase in Escherichia Coli K-12: Involvement of chlC, chlE, and chlG Loci. J. Bacteriol..

[39] Guilbault G.G., Brignac P.J., Juneau M. (1968). New substrates for the fluorometric determination of oxidative enzymes. Anal. Chem..

[40] Ran F.A., Hsu P.D., Wright J., Agarwala V., Scott D.A., Zhang F. (2013). Genome engineering using the CRISPR-Cas9 system. Nat. Protoc..

[41] Struhl K. (1991). Subcloning of DNA Fragments. Curr. Protoc. Mol. Biol..

[42] Ladner C.L., Yang J., Turner R.J., Edwards R.A. (2004). Visible fluorescent detection of proteins in polyacrylamide gels without staining. Anal. Biochem..

